# Annexin-V positive extracellular vesicles level is increased in severe COVID-19 disease

**DOI:** 10.3389/fmed.2023.1186122

**Published:** 2023-06-02

**Authors:** Valentine Jacob, Alexis Lambour, Benjamin Swinyard, Yoann Zerbib, Momar Diouf, Simon Soudet, Etienne Brochot, Isabelle Six, Julien Maizel, Michel Slama, Nicolas Guillaume

**Affiliations:** ^1^Department of Human Biology Center, Amiens University Medical Center, Amiens, France; ^2^EA HEMATIM 4666, Jules Verne University of Picardie, Amiens, France; ^3^Department of Medical Intensive Care Unit, Amiens University Medical Center, Amiens, France; ^4^Department of Statistics, Amiens University Medical Center, Amiens, France; ^5^Department of Vascular Medicine, Amiens University Medical Center, Amiens, France; ^6^AGIR Research Unit, Jules Verne University of Picardie, Amiens, France; ^7^UR 7517 UPJV, Pathophysiological Mechanisms and Consequences of Cardiovascular Calcifications (MP3CV), Jules Verne University of Picardie, Amiens, France

**Keywords:** extracellular vesicles, microparticles, COVID-19, thrombosis, intensive care unit

## Abstract

**Objectives:**

To evaluate extracellular vesicles levels in a cohort of SARS-CoV-2’s patients hospitalized in an intensive care unit with and without COVID-19 associated thromboembolic events.

**Methods:**

In this study, we aim to assess endothelial and platelet membrane-derived extracellular vesicles levels in a cohort of SARS-CoV-2 patients with and without COVID-19-associated thromboembolic events who were hospitalized in an intensive care unit. Annexin-V positive extracellular vesicles levels were prospectively assessed by flow cytometry in one hundred twenty-three critically ill adults diagnosed with acute respiratory distress syndrome associated with a SARS-CoV-2 infection, ten adults diagnosed for moderate SARS-CoV-2 infection and 25 healthy volunteers.

**Results:**

On our critically ill patients, thirty-four patients (27.6%) had a thromboembolic event, Fifty-three (43%) died. Endothelial and platelet membrane-derived extracellular vesicles were drastically increased in SARS-CoV-2 patients hospitalized in the ICU compared to healthy volunteers. Moreover a slighty higher small/large ratio for platelets membrane-derived extracellular vesicles in patients was linked to thrombo-embolic events.

**Conclusion:**

A comparison between total annexin-V positive extracellular vesicles levels in severe and moderate SARS-CoV-2 infection and healthy controls showed a significant increase in patients with severe infection and their sizes could be considered as biomarkers of SARS-CoV-2 associated thrombo-embolic events.

## Introduction

Coronavirus 2019 (COVID-19) infection can lead to severe acute respiratory syndrome due to coronavirus 2 (SARS-CoV-2). This disease is also associated with cardiovascular complications such as thrombo-embolic events (1). During the course of the disease, some macro or micro thromboembolic events, and disseminated intravascular coagulation have been described. Indeed, viral diseases can damage the endothelium in many ways. Endothelial cells are key regulators of vasomotricity, oxidative stress, coagulation, inflammation and are an important mediator of atherosclerosis and vascular disease (2). The virus can bind the angiotensin-converting enzyme 2 (ACE-2) receptor expressed notably by type 2 alveolar cells, bronchial epithelial cells, and endothelial cells (1). Viral elements have been found inside endothelial cells, and associated to an accumulation of inflammatory cells, leading to endothelial and inflammatory cell death (3).

Extracellular vesicles (EVs) are lipid-bound vesicles secreted into the extracellular space. EVs are heterogeneous membranous vesicles with different sizes, functions and origins. They include exosomes, microvesicles, and apoptotic bodies, which contain lipids, nucleic acids and proteins (4). Exosomes are released continuously from cells, whereas microvesicles and apoptotic bodies are released predominantly by activated or apoptotic cells. EVs are generated by plasma membrane blebbing with externalization of phosphatidylserine. Indeed, in physiological conditions, phosphatidylserine is usually located only in the cytoplasmic leaflet (4). EVs can transfer these molecules to target cells in a communication system between neighboring and distant cells and tissues. EVs secreted by endothelial cells have some effects such as antioxidant effects by including for example glutathione transferases and peroxidases which may contribute to maintaining blood plasma redox state (5, 6), effects on endothelial cell activation through for example miRNA (7), role in oxidative stress-mediated dysfunction of endothelial cells. EVs from platelets play a role in coagulation, linked to the surface exposure of negatively charged phospholipids (8).

In this study, we aim to assess endothelial and platelet membrane-derived extracellular vesicles levels (eEVs and pEVs, respectively) in a cohort of SARS-CoV-2 patients with and without COVID-19-associated thromboembolic events who were hospitalized in an intensive care unit.

## Materials and methods

### Patients

Consecutive patients (*n* = 131) referred to the medical intensive care medicine department in the Amiens University Hospital at, France between February 2020 and October 2021 who were diagnosed with acute respiratory distress syndrome associated with a SARS-CoV-2 infection (severe disease) were prospectively enrolled in this study. Severe disease was defined by at least one visceral failure (most often respiratory failure) requiring transfer to ICU. Eight patients were excluded in relation to a negative or questionable COVID-19 test (RT-PCR). Only data from 123 patients were analyzable for our study. This study “Thromboembolic events in hospitalized patients with COVID-19 serious acute pneumopathy (THROMBOCOVID1)” was reviewed and approved by the Institutional Review Bord (IRB) for the local Ethics Committee of Amiens Nord-Ouest II and registered on clinicaltrials.gov with the identifier NCT04377490. The need for IRB approval and informed consent was waived on May 4, 2020. All procedures were followed in accordance with the ethical standards of the responsible committee on institutional human experimentation and with the Helsinki Declaration of 1975. As some patients were recruited during the vaccination campaign, 8 patients were vaccinated (4 with one dose, 2 with two doses, 2 with three doses).

We also selected 10 consecutive patients referred to our medicine department, for moderate SARS CoV-2 infection who did not meet the criteria used to define patients with severe disease (no organ support, no invasive ventilation, no vasoactive drugs, no visceral failure). All patients or next-of-kin signed an informed consent.

### Healthy individuals

Healthy volunteers (*n* = 25) were selected from a bone marrow donation center. None of the volunteers took any drugs at that time or had chronic disease. They had no clinical signs of viral infection. COVID-19 tests were performed in the week within the selection and were negative. All participants signed an informed consent.

### Investigations

All patients underwent a complete clinical examination, with laboratory and morphological measurements at Day 0, Day 7, Day 14, Day 21, and Day 28. Examinations included Doppler ultrasound in the case of a suspected venous thrombosis and a pulmonary CT angiography in the case of a suspected pulmonary embolism.

### Endothelial and platelet membrane-derived extracellular vesicles (eEVs and pEVs)

Samples for EVs measurement were collected at Day 0, Day 7, Day 14, Day 21, and Day 28 according to the patients’ availability. EVs were isolated from whole blood using a common differential centrifugation assay described in the literature (9). The citrated tubes were quickly centrifuged (2,500 × g for 15 min at room temperature). Supernatant was collected and platelet poor plasma was stored at −80°C. After thawing, we performed a second centrifugation of the supernatant (25,000 × g for 30 min at room temperature) and this platelet poor plasma was used to study EVs concentration and phenotype.

EV detection was then performed on thawed samples using a Cytoflex cytometer (Beckman Coulter Life Sciences, Villepinte, France). Annexin V-BV510 (Brillant Violet 510) as the EV marker (BioLegend, Paris, France), anti-CD41-FITC (IM0649U) as platelets marker and anti-CD144-PE (A07481) as endothelial marker (Beckman Coulter Life Sciences, Villepinte, France) were used for MP detection. Megamix-plus FSC beads (BioCytex, Marseille, France) calibrated from 0.1 to 0.9 μm were used to define an analysis window consistent with the size of EVs. EVs were quantified using EV Count Beads (BioCytex, Marseille, France): [EV counts × (EV-count beads)]/EV count beads counted. In order to discriminate EVs and aggregates, we set the signal detection at peak height (V-SSC-H and FSC-H) and peak width.

### Statistical analysis

Categorical variables were presented as frequencies and continuous variables were presented as the median and 25th to 75th percentiles. Statistical analyses were performed using the nonparametric Mann–Whitney and Kruskal–Wallis tests for testing the hypothesis that the distributions of each of two groups was close for quantitative variables and Fisher’s exact test for frequencies to assess the existence of statistical differences between groups. For EVs follow-up with this measurement at more than two time points, we used a repeated measures Anova test. A *p*-value <0.05 was considered statistically significant.

## Results

### Demographics, clinical characteristics, and biological parameters of the study population

One hundred twenty-three patients were included in this study. The clinical and biological characteristics of all included patients are listed in Table 1. Mean age was 68 years, age range 58.5–72 years, 88 (71%) were male. Ninety patients (73%) required mechanical ventilation for a median of 18 days. Thirteen patients (10.5%) required extracorporeal circulation, 53 patients (43%) died. Thirty-four patients (27.6%) had a thromboembolic event: 25 deep vein thrombosis, 2 arterial thrombosis, 15 pulmonary embolisms (including the 6 associated with deep vein thrombosis). Four thromboses were detected on foreign material (2 on central catheter, one on extracorporeal circulation cannula, one on arterial cannula).

**Table 1:** Demographic characteristics of patients with severe form of COVID-19.

**Patients (*n* = 123)**	
Age years, median (range)	67 (58.5–72)
Male	35 (71.5%)
Female	54 (28.5%)
BMI, mean (SD)	29.4 (25.3–33.6)
**Comorbidities**	
Arterial hypertension	60 (49%)
Auricular fibrillation	6 (5%)
Ischemic cardiopathy	18 (14.5%)
Thromboembolism	8 (6.5%)
Oral anticoagulants	14 (11%)
Diabetes	37 (30%)
Renal impairment	18 (14.5%)
Chronic dialysis	2 (1.5%)
Cancer	17 (14%)
Obstructive sleep apnea	7 (5.5%)
Chronic obstructive pulmonary disease	5 (4%)
BMI > 30	57 (46%)
**Follow-up in the intensive care medicine department**	
Sepsis-related organ failure assessment (SOFA), mean	5 (3–11)
Stay in the intensive unit (meantime in days)	19 (8.5–29.5)
Endotracheal intubation	90 (73%)
Invasive ventilation, meantime in days	18 (10–33.5)
Extracorporeal membrane oxygenation, number of patients	13 (10.5%)
Thromboembolic events	34 (27.6%)
Pulmonary embolism	15 (12%)
Dexamethasone use	109 (88.5%)
Tocilizumab use	8 (6.5%)
Death	53 (43%)

### Endothelial and platelet membrane-derived extracellular vesicles are drastically increase in SARS-CoV-2 patients hospitalized in an intensive care unit compared to healthy volunteers

**Figure 1:**
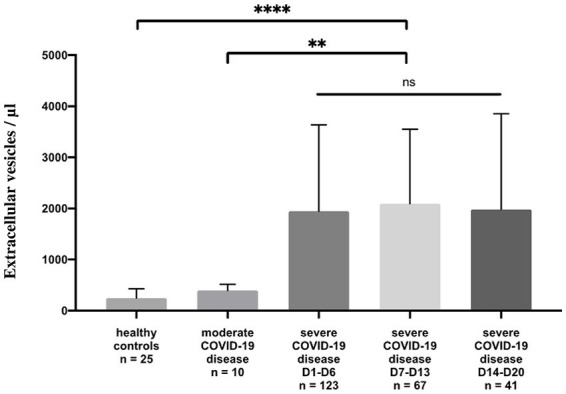
Annexin-V positive EV levels in healthy controls and COVID-19 patients. Plasma levels (/μL) of annexin-V positive EVs in healthy controls compared to moderate and severe COVID-19 disease. For severe disease, three measure points are performed: Day 1 to Day 6, Day 7 to Day 13, and Day 14 to Day 20, the duration of the ICU stay.

**Figure 2:**
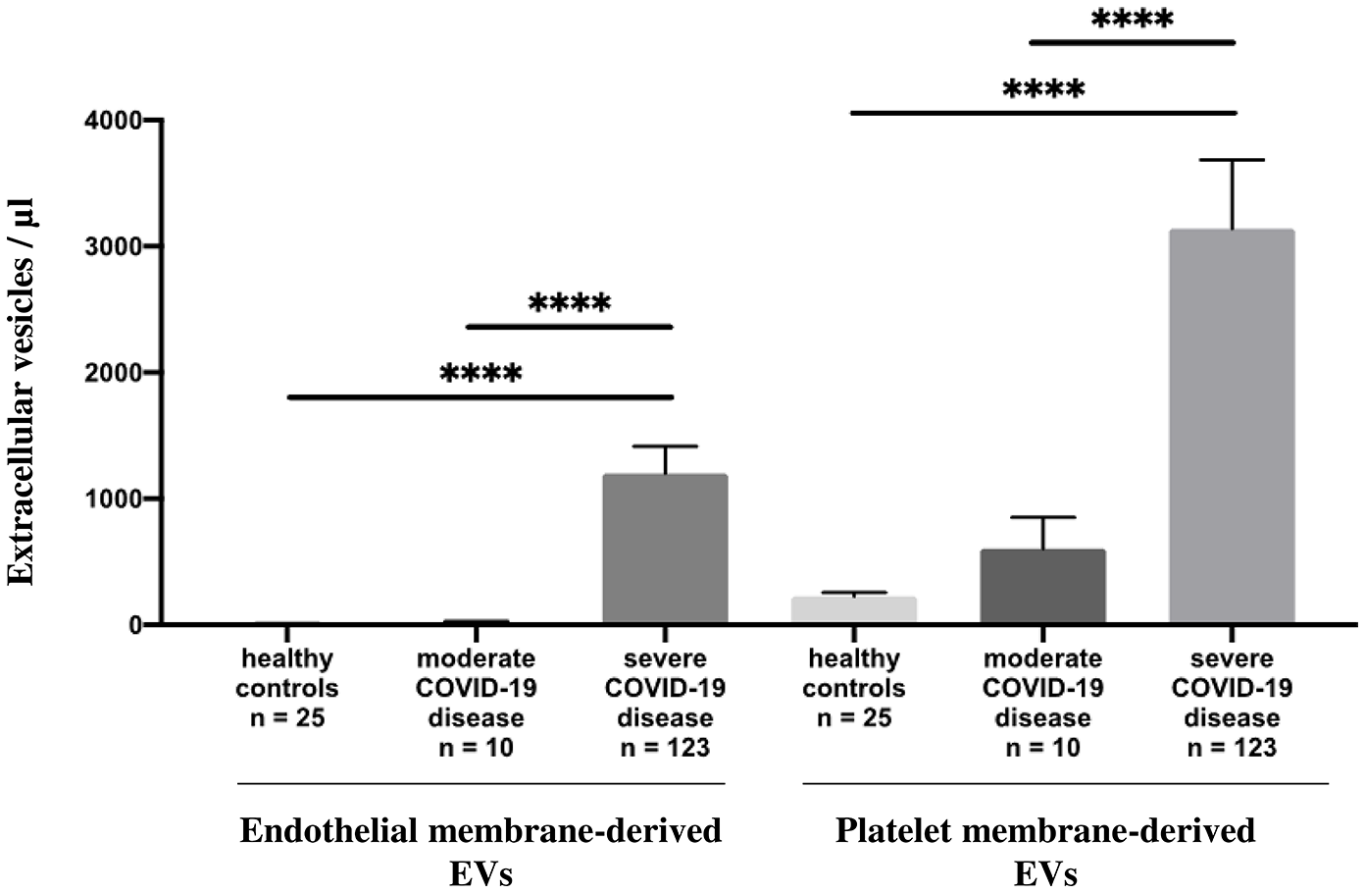
Annexin-V positive EV levels in healthy controls and COVID-19 patients. Plasma levels (/μL) of annexin-V positive endothelial and platelet membrane-derived EVs in healthy controls compared to moderate and severe COVID-19 disease.

Circulating annexin-V positive EV subsets were quantified by sensitive flow cytometry using annexin-V forward scatter gating to identify annexin-V positive EVs of interest by size. This allows for the discrimination of small and large EVs (Supplementary Figure S1). They were drastically increased in patients with severe disease [median 1944/μL, 25th to 75th percentile (837.5–3595)] compared with those of patients with moderate disease [median 410.5/μL, 25th to 75th percentile (275.5–995.5)] *p* = 0.004 and healthy volunteers [median 247/μL, 25th to 75th percentile, (157.5–429.5)] *p* < 0.0001 (Figure 1; Supplementary Table S1) and this continued for the duration of the ICU stay (follow-up Day 1 to Day 28). Platelet (pEVs) and endothelial-derived (eEVs) extracellular vesicles were also increase in patients with severe disease compared with moderate disease and healthy controls (Figure 2; Supplementary Table S1). By analyzing annexin-V positive EVs with forward scatter, we identified several profiles of EVs according to their small and large sizes. Small and large total EVs were increased in patients with severe disease, especially pEVs (Supplementary Table S1).

### Endothelial and platelet membrane-derived extracellular vesicles levels are not linked to thromboembolic events in SARS-CoV-2 in-patients

**Figure 3:**
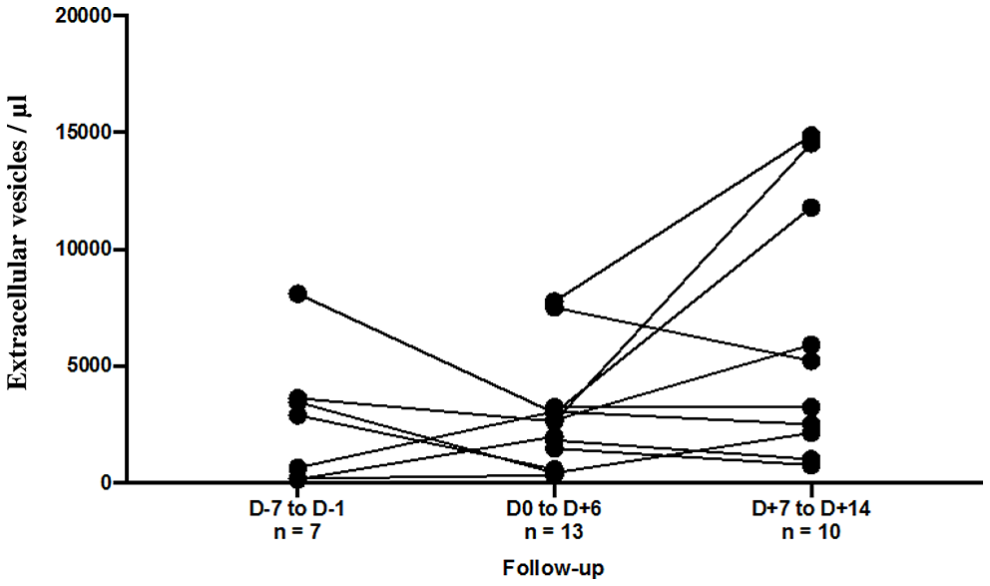
Follow-up of annexin-V positive EV levels (/μL) according to the thromboembolic event. For 6 patients, EV levels were assessed at Day 7 to Day 1 before and at Day 1 to Day 6 after the thromboembolic event. For 10 patients, EV levels were assessed at Day 1 to Day 6 and at Day 7 to Day 14 after the thromboembolic event.

To better understand the degree to which EVs are associated with severe disease, we compared the data between severe disease with and without thromboembolic events, *n* = 34 and *n* = 89, respectively. Unfortunately, none of the studied EV subsets showed any significant difference between these two populations (total, endothelial and platelet-derived small and large size EVs). However, we observed a trend of a slightly higher small/large ratio for total EVs (*p* = 0.04) and pEVs (*p* = 0.02) in patients with thrombo-embolic events (Supplementary Table S2). For a limited number of patients, we established the follow-up of annexin-V positive EV levels before and after the thrombo-embolic events (*n* = 13). These events did not appear to induce a significant and reproductible change in the levels of our annexin-V positive EV subsets (Figure 3). This distinction was difficult to study because of the small number of patients who could be followed up for more than two weeks.

## Discussion

Circulating annexin-V positive EVs were quantified by sensitive flow cytometry in a cohort of patients diagnosed with acute respiratory distress syndrome associated with a SARS-CoV-2 infection referred to an intensive care medicine department. Our data demonstrate a higher total annexin-V positive EV level in severe disease compared to moderate SARS-CoV-2 infection and healthy controls, with a significant increase in eEVs and particularly in pEVs.

In our study, we have included our patients over a large period of time including several pandemic waves. However the virus-variant status did not appear to be a factor of heterogeneity for our cohort. Indeed a previous study showed high similar counts especially of pEVs in two SARS-CoV-2 positive cohorts enrolled during both the first and the second pandemic waves (10).

In several studies, pEVs had already been found in association with viral infections such as the H1N1 virus (11), HIV (12), and dengue virus (13). They point to EVs and pEVs as potential biomarkers in COVID-19. Indeed, increased levels of circulating pEVs have been observed in patients with SARS-CoV-2 infection (10, 14, 15). In the same way, high plasma concentrations of eEVs were also observed (15). In particular, patients with severe disease (intubated group) exhibited increased eEVs concentrations and mean size compared to an uninfected group. These eEVs also induced endothelial expression of pro-adhesive proteins (16), in line with a procoagulant and hyperinflammation setting. Indeed, the most severe forms of COVID-19 involve endothelial damage, clot formation, microthrombi, and multiple organ failure, suggesting a central role of the vascular endothelium (17). However, in our study, although we demonstrated a clear increase in total EV levels in severe COVID-19 infection, we failed to distinguish, in this way, thromboembolic events in this specific population. Campello et al. (15) showed that baseline levels of pEVs were significantly associated with the development of thromboembolic events with a low OR (1.07) in a cohort of mild and moderate COVID-19 infection. In contrast, Guervilly et al. (18) did not show differences in EV subsets between the moderate and severe forms of COVID-19, but the activity of tissue factor linked to these EVs was significantly increased in patients with severe disease, but also in patients with symptomatic clinical thromboembolic events within 28 days after sampling. Another study showed that plasma procoagulant extracellular vesicles (tissue factor activity) were elevated ∼nine-fold in severe COVID-19 patients (19). These data could explain the strong procoagulant imbalance in severe infection not only linked to the levels of EVs, but also by a high level of tissue factor activity. The systemic inflammation in severe COVID-19 disease leads to a wide range of hemostatic derangements in which the study of EVs is of interest. Moreover, we showed a slightly higher small/large ratio, especially for pEVs (*p* = 0.02) in patients with thrombo-embolic events. This observation could be linked with previous studies showing that stimuli can activate platelets, resulting in the genesis of pEVs. The quantity, the structure and the size of the pEVs clearly depend on the type of activation. After activation, platelets undergo a conformational change, releasing effector molecules and EVs (20). Then, collagen and ADP activation induced spherical and smooth EVs production. Moreover with collagen or thrombin activation, many more EVs are produced. But, in case of thrombin activation, MPs are smaller, both spherical and elongated form (21, 22). Surface exposure of phosphatidylserine can explain the procoagulant effects of pEVS. Moreover, pEVs were more procoagulant when they were activated by a combination of collagen and thrombin (8). In COVID-19 infection, platelets internalized SARS-CoV-2 virions leading to EVs release (23). However, platelets activation was not directly induced by SARS-CoV-2 or purified spike but rather to the release of active tissue factor by infected cells (24).

## Conclusion

Total annexin-V EV levels were increased in severe disease compared to moderate SARS CoV-2 infection and healthy controls, with a significant increase in eEVs, and particularly pEVs. In patients with thromboembolic events, although pEV and eEV levels were not linked to thromboembolic events, we nevertheless showed a slightly higher small/large ratio, especially for pEVs which seems to be an interesting way to explore in the link between severe COVID disease and thrombo-embolic events.

## Data availability statement

The original contributions presented in the study are included in the article/Supplementary material, further inquiries can be directed to the corresponding authors.

## Ethics statement

The studies involving human participants were reviewed and approved by Institutional Review Bord (IRB) for the local Ethics Committee of Amiens Nord-Ouest II and registered on clinicaltrials.gov with the identifier NCT04377490. The patients/participants provided their written informed consent to participate in this study.

## Author contributions

VJ, AL, BS and YZ: acquisition of data, analysis and interpretation of data, revising the article critically for important intellectual content, final approval of the version to be submitted. MD: analysis and interpretation of data, final approval of the version to be submitted. SS, EB, IS and JM: acquisition of data, final approval of the version to be submitted. MS and NG: conception and design of the study, interpretation of data, drafting the article and revising it critically for important intellectual content, and final approval of the version to be submitted.

## Funding

This work was funded by the Amiens University Medical Center (Amiens, France) as part of the French government’s program for hospital-based clinical research.

## Conflict of interest

The authors declare that the research was conducted in the absence of any commercial or financial relationships that could be construed as a potential conflict of interest.

## Publisher’s note

All claims expressed in this article are solely those of the authors and do not necessarily represent those of their affiliated organizations, or those of the publisher, the editors and the reviewers. Any product that may be evaluated in this article, or claim that may be made by its manufacturer, is not guaranteed or endorsed by the publisher.
