# Supplementary material for: Annexin-V positive extracellular vesicles level is increased in severe COVID-19 disease
Source: Front Med (Lausanne). 2023 Jun 2;10:1186122. doi: 10.3389/fmed.2023.1186122 (PMC10272544; doi:10.3389/fmed.2023.1186122)
Supplement: Supplementary file 1 [file Data_Sheet_1.PDF]

Supplemental S1 : Levels of Annexin-V positive EVs (/µl) according to patients status (severe and moderate Covid-19 disease ; healthy controls)

	**Severe disease COVID-19 n = 123**	**Healthy controls n = 25**	**p value**	**Moderate disease COVID-19 n = 10**	**p value**
**Annexin-V positive EVs (median and interquartile)**					
**Total (/µl)**	1944 [837,5 – 3595]	247 [157,5 – 429,5]	< **0,0001**	410,5 [275,5 – 995,5]	**0,004**
**Small EVs (/µl)**	1037 [3832 – 2253]	83 [58,5 – 181,5]	< **0,0001**	105 [77 – 182]	**0,0003**
**Large EVs (/µl)**	779 [318,3 – 1431]	147 [73,5 – 284,5]	< **0,0001**	237 [187 – 360]	0,4
**Ratio Small/Large**	1,3 [0,8 – 2,2]	0,7 [0,4 – 1,35]	**0,002**	0,5 [0,2 – 0,8]	**0,0004**
**Annexin-V positive endothelial-derived EVs (median and interquartile)**					
**Total (/µl)**	149,5 [48,5 – 790,5]	8 [4,5 – 10,5]	< **0,0001**	17 [9 – 30]	**0,0006**
**Small EVs (/µl)**	162 [37,75 – 781,3]	4 [2 – 5]	< **0,0001**	16,5 [12,75 – 23,5]	< **0,0001**
**Large EVs (/µl)**	50 [14,75 – 238,5]	3 [1 – 4,5]	< **0,0001**	1 [0 – 3,0]	< **0,0001**
**Annexin-V positive platelet-derived EVs (median and interquartile)**					
**Total (/µl)**	1352 [500 – 2703]	194 [108,5 – 337]	< **0,0001**	291 [140 – 463]	**0,0002**
**Small EVs (/µl)**	538,5 [191,8 – 1250]	39 [33 – 61,5]	< **0,0001**	86 [24 – 121]	**0,01**
**Large EVs (/µl)**	642 [229,8 – 1444]	109 [45,5 – 215,5]	< **0,0001**	138 [83 – 324]	< **0,0001**

Supplemental S2

	**Severe disease COVID-19 without thrombo-embolic event**<br>n = 89	**Severe disease COVID-19 with thrombo-embolic event**<br>n = 34	p
**Annexin-V positive EVs (median and interquartile)**			
**Total (/µl)**	1853 [791 – 3604]	2635 [851 – 3874]	0,2
**Small EVs (/µl)**	1008 [389 – 2222]	1617 [529,5 – 2276]	0,2
**Large EVs (/µl)**	805 [313 – 1575]	782 [322 – 1238]	0,3
**Ratio Small/Large**	1,2 [0,7 – 2,3]	2,1 [1,2 – 3,05]	**0,04**
**Annexin-V positive endothelial-derived EVs (median and interquartile)**			
**Total (/µl)**	152 [54 – 858]	720 [34 – 1284]	0,2
**Small EVs (/µl)**	112 [39 – 545]	325 [21,5 – 865,5]	0,2
**Large EVs (/µl)**	60 [16 – 233]	159 [12,5 – 338]	0,2
**Ratio Small/Large**	2,4 [1,7 – 3,5]	3,1 [1,7 – 3,4]	0,4
**Annexin-V positive platelet-derived EVs (median and interquartile)**			
**Total (/µl)**	1281 [541 – 2715]	1866 [642,5 – 2599]	0,2
**Small EVs (/µl)**	496 [191 – 1208]	892 [339 – 1526]	0,09
**Large EVs (/µl)**	655 [246 – 1491]	757 [303,5 – 1193]	0,4
**Ratio Small/Large**	0,7 [0,4 – 1,2]	1,2 [0,7 – 2,15]	**0,02**
